# Enhanced Immunogenicity of Stabilized Trimeric Soluble Influenza Hemagglutinin

**DOI:** 10.1371/journal.pone.0012466

**Published:** 2010-09-01

**Authors:** William C. Weldon, Bao-Zhong Wang, Maria P. Martin, Dimitrios G. Koutsonanos, Ioanna Skountzou, Richard W. Compans

**Affiliations:** Department of Microbiology and Immunology and Emory Vaccine Center, Emory University School of Medicine, Atlanta, Georgia, United States of America; University of Georgia, United States of America

## Abstract

**Background:**

The recent swine-origin H1N1 pandemic illustrates the need to develop improved procedures for rapid production of influenza vaccines. One alternative to the current egg-based manufacture of influenza vaccine is to produce a hemagglutinin (HA) subunit vaccine using a recombinant expression system with the potential for high protein yields, ease of cloning new antigenic variants, and an established safety record in humans.

**Methodology/Principal Findings:**

We generated a soluble HA (sHA), derived from the H3N2 virus A/Aichi/2/68, modified at the C-terminus with a GCN4pII trimerization repeat to stabilize the native trimeric structure of HA. When expressed in the baculovirus system, the modified sHA formed native trimers. In contrast, the unmodified sHA was found to present epitopes recognized by a low-pH conformation specific monoclonal antibody. We found that mice primed and boosted with 3 µg of trimeric sHA in the absence of adjuvants had significantly higher IgG and HAI titers than mice that received the unmodified sHA. This correlated with an increased survival and reduced body weight loss following lethal challenge with mouse-adapted A/Aichi/2/68 virus. In addition, mice receiving a single vaccination of the trimeric sHA in the absence of adjuvants had improved survival and body weight loss compared to mice vaccinated with the unmodified sHA.

**Conclusions/Significance:**

Our data indicate that the recombinant trimeric sHA presents native trimeric epitopes while the unmodified sHA presents epitopes not exposed in the native HA molecule. The epitopes presented in the unmodified sHA constitute a “silent face” which may skew the antibody response to epitopes not accessible in live virus at neutral pH. The results demonstrate that the trimeric sHA is a more effective influenza vaccine candidate and emphasize the importance of structure-based antigen design in improving recombinant HA vaccines.

## Introduction

Influenza virus is one of the most common causes of serious respiratory illness. Since the beginning of the 2009–2010 influenza season, the CDC has reported that all subtyped influenza A viruses isolated from hospitalizations were the novel 2009 H1N1 virus [1]. Monovalent H1N1 vaccines have been distributed in the United States; however, due to lower than expected production levels a vaccine shortage occurred. Influenza vaccine production in eggs requires the generation of high-yield egg growth reassortant viruses and a large supply of embryonated chicken eggs, and the vaccine has potential safety concerns in individuals with egg allergies [2], [3], [4], [5]. For these reasons, efforts have been made to develop alternative vaccine production methods. Production of influenza proteins in recombinant expression systems is one such approach; recombinant HA vaccine has been produced in bacterial, plant, mammalian, and recombinant baculovirus (rBV) expression systems. The rBV expression system has several advantages over traditional egg-based vaccine production techniques including the high levels of expression of recombinant protein[6], rapid scaling of production to meet high demands[2], an established safety record[2], [5], [7], and more rapid production of new vaccines in response to antigenic shift or drift[2], [8]. One disadvantage to recombinant HA vaccines is that other viral proteins are not present such as neuraminidase or M2.

Proteins produced in the rBV system use the same glycosylation sites as mammalian proteins. However, in the insect cell system the major carbohydrate moiety is high-mannose carbohydrate while mammalian systems have elongated carbohydrates with the common high-mannose intermediate[6]. Previous studies have demonstrated that the recombinant HA produced in insect and mammalian expression systems did not have significantly altered receptor binding[9]. Despite the difference in glycosylation, the immunogenicity of insect derived HA is comparable to mammalian expressed HA[10].

The seasonal influenza vaccine is trivalent, containing two type A influenza strains (H1N1 and H3N2) and one type B influenza strain. The major component of influenza vaccine is the viral hemagglutinin (HA), a trimeric type I transmembrane protein consisting of two chains, HA1 and HA2, linked by inter-chain disulfide bonds[11]. During the influenza virus life cycle, HA functions to bind terminal sialic acid residues on host cell glycoproteins, initiating receptor mediated endocytosis and allowing the virus to enter the cell in an endosome. Once internalized, HA mediates fusion of viral and host membranes via a low pH- induced structural conformation change [11], [12], [13].

Currently, there are 16 distinct antigenic subtypes of HA in influenza A viruses[14]. There are two major mechanisms enabling influenza A viruses to evade the immune response and spread more rapidly amongst a population, antigenic drift and antigenic shift. Antigenic drift is a result of point mutations in the HA and NA genes which are under selective pressure from the host antibody response. Antigenic shift is the term used to describe the emergence of novel viruses by the genetic reassortmant of viral RNA segments.[11], [15]

Because the HA is the most immunologically important component of influenza vaccines, we generated a system for producing a protein subunit vaccine using a rBV-derived soluble influenza HA modified with a trimerization heptad repeat derived from the transcription factor GCN4 [16], [17], [18], to stabilize the native trimeric structure of the HA protein. We determined the effect of this stabilization on the immunogenicity of the protein vaccine and its ability to induce a protective immune response to challenge with homologous virus in mice primed and boosted with a low dose of vaccine without adjuvants.

## Results

### Recombinant baculovirus derived Aichi/2/68 soluble HA is expressed as the HA0 precursor

**Figure 1:**
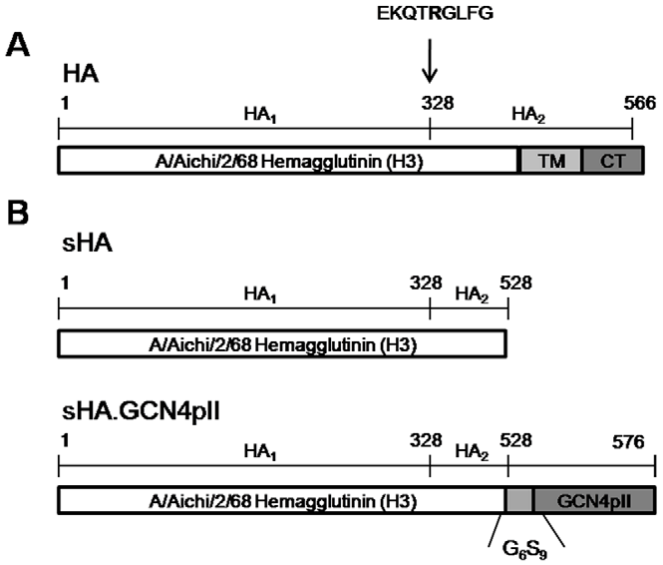
Recombinant soluble HA constructs. **A.** Full length H3N2 A/Aichi/2/68 hemagglutinin precursor (HA0). TM  =  transmembrane domain, CT  =  cytosolic domain. Cleavage site at R328 is indicated by arrow. **B.** Soluble H3N2 A/Aichi/2/68 HA constructs. Soluble H3N2 HA was generated by eliminating the transmembrane (TM) and cytosolic (CT) domain. The modified trimeric soluble H3N2 HA was generated by fusing the GCN4pII trimerization repeat to the C-terminus of HA with a short peptide linker (G6S9).

**Figure 2:**
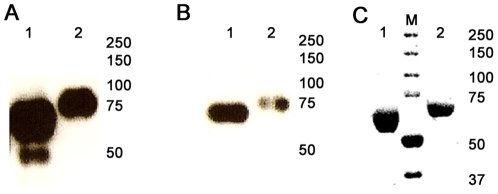
Recombinant H3N2 A/Aichi/2/68 is expressed as the hemagglutinin precursor (HA0). **A,B.** Western blot analysis of SDS-PAGE separated sHA (Lane 1) and sHA.GCN4pII (Lane 2) using polyclonal sera or anti-histidine tag monoclonal antibody (primary antibody). Protein bands were developed using goat anti-mouse HRP (secondary antibody) and ECL Plus (GE Healthcare). **C.** Coomassie blue stain of sHA (Lane 1) and sHA.GCN4pII (Lane 3) after nickel-bead column purification. Lane 2 - molecular weight marker. Each lane was loaded with 1 µg of recombinant protein.

Diagrams of recombinant baculovirus expression constructs for the soluble HA derived from the H3N2 virus A/Aichi/2/68 with the GCN4pII modification are shown in Figure 1. Sf9 insect cells expressed and secreted ∼8 µg of purified recombinant protein per mL of Sf9 cell culture. The sHA and sHA.GCN4pII proteins migrated on SDS-PAGE as single polypeptides of approximately 70 and 75 kDa proteins, respectively, indicating that the recombinant proteins were expressed as HA precursors (HA0). The larger molecular weight of the sHA.GCN4pII protein indicates that the GCN4pII modification was added to the C-terminus of the H3N2 HA protein. Western blot analysis of the recombinant HA proteins using reference sera from A/Aichi/2/68 infected mice indicates that the recombinant proteins are antigenically similar to the viral HA (Figure 2A) and the anti-His monoclonal antibody binding demonstrates that the His-tag was included in the protein (Figure 2B)(Lane 1  =  sHA, Lane 2  =  sHA.GCN4pII). Coomassie blue staining after SDS-PAGE separation of the sHA and sHA.GCN4pII following nickel-bead chromatography showed a single band corresponding to the recombinant proteins indicating a relatively high level of purity (Figure 2C). Furthermore, both recombinant proteins are produced as the HA0 precursor at a high yield in the baculovirus expression system.

**Figure 3:**
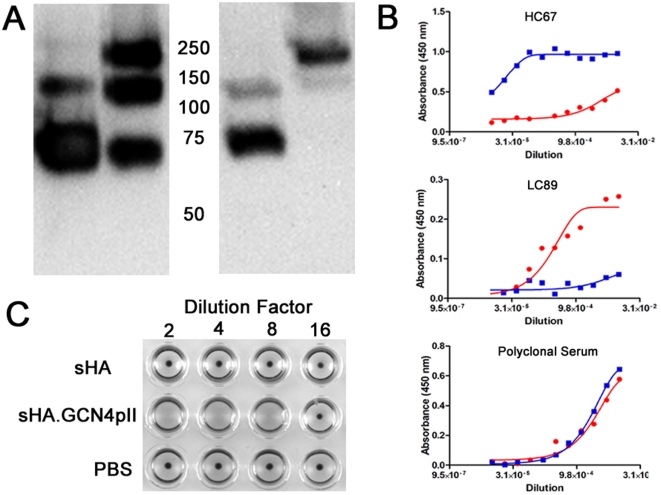
GCN4pII modification stabilizes the trimeric structure of A/Aichi/2/68 soluble HA. **A.** BS_3_ crosslinking was performed as described in Materials and Methods. Lanes 1 and 2 – sHA, Lanes 3 and 4 – sHA.GCN4pII. Lane 1 and 3, no BS3, Lanes 2 and 4, 3 mM BS3. Trimeric HA corresponded to a band of ∼220 kDa, dimeric HA corresponds to a band of ∼140 kDa, and monomeric HA corresponded to a band of ∼75 kDA Western blot primary antibody, anti-histidine monoclonal antibody. **B.** Sandwich ELISA. 20 µg of sHA (red circle) or sHA.GCN4pII (blue square) were captured using guinea pig anti-A/Aichi/2/68 then binding affinity of HC67, LC89 or polyclonal sera was detected by absorbance at 450 nm. **C.** Hemagglutination test using 1 µg of recombinant protein (sHA or sHA.GCN4pII) or PBS. Proteins were diluted in PBS and incubated at room temperature for 30 minutes with 0.05% chicken red blood cells (washed).

To determine the oligomeric structure of the recombinant proteins, cross-linking was performed using BS3, a water soluble cross-linker approximately 11 angstrom long which reacts with primary amines thus covalently linking proteins together. Multimeric proteins exposed to this crosslinker will have each subunit crosslinked together, allowing them to be analyzed on denaturing gels for Western blot analysis [19], [20]. Using this approach, we determined the oligomeric structure of the recombinant soluble HA with and without the GCN4pII modification. Separation of reaction products on gradient SDS-PAGE and Western blot analysis indicates that the sHA is observed as a mixture of trimeric (∼210 kDa), dimeric (∼140 kDa), and monomeric proteins (∼70 kDa)(Figure 3A – Lane 1,2). However, the modification of the sHA protein at the C-terminus with the GCN4pII trimerization repeat stabilized the trimeric structure of the secreted recombinant protein (∼220 KDa)(Figure 3A – Lane 3,4).

In addition to analysis by chemical crosslinking of the recombinant proteins, monoclonal antibody binding was used to determine the conformation of the recombinant soluble HA. Antibody binding by sandwich ELISA indicates that the trimeric A/Aichi/2/68 HA specific monoclonal antibody HC67 bound to sHA.GCN4pII with a higher affinity compared to the sHA, indicating that trimeric HA was the predominant form in the modified soluble HA (Figure 3B). This supports our findings using crosslinking of the recombinant proteins. The low-pH A/Aichi/2/68 HA specific monoclonal antibody LC89 bound to the unmodified sHA with a higher affinity than the trimeric sHA indicating that the unmodified but not the modified trimeric sHA exhibits epitopes which are exposed in the low-pH conformation.

Low-pH treated bromelain-treated HA (BHA) forms rosettes in solution via hydrophobic interactions between the fusion peptide of adjacent BHA, allowing the protein to agglutinate red blood cells [20], [21]. We compared recombinant sHA and sHA.GCN4pII in a hemagglutination assay, and found that the sHA.GCN4pII protein was able to agglutinate chicken RBC while the sHA was not (Figure 3C). This demonstrates that the modified protein folds into the native structure of the HA and has retained the ability to bind sialic acid receptors on red blood cells, causing agglutination. Therefore, modification of the H3N2 Aichi HA at the C-terminus with the GCN4pII repeat does not prevent the receptor binding activity of the trimeric protein.

### sHA.GCN4pII induces a robust humoral immune response

**Figure 4:**
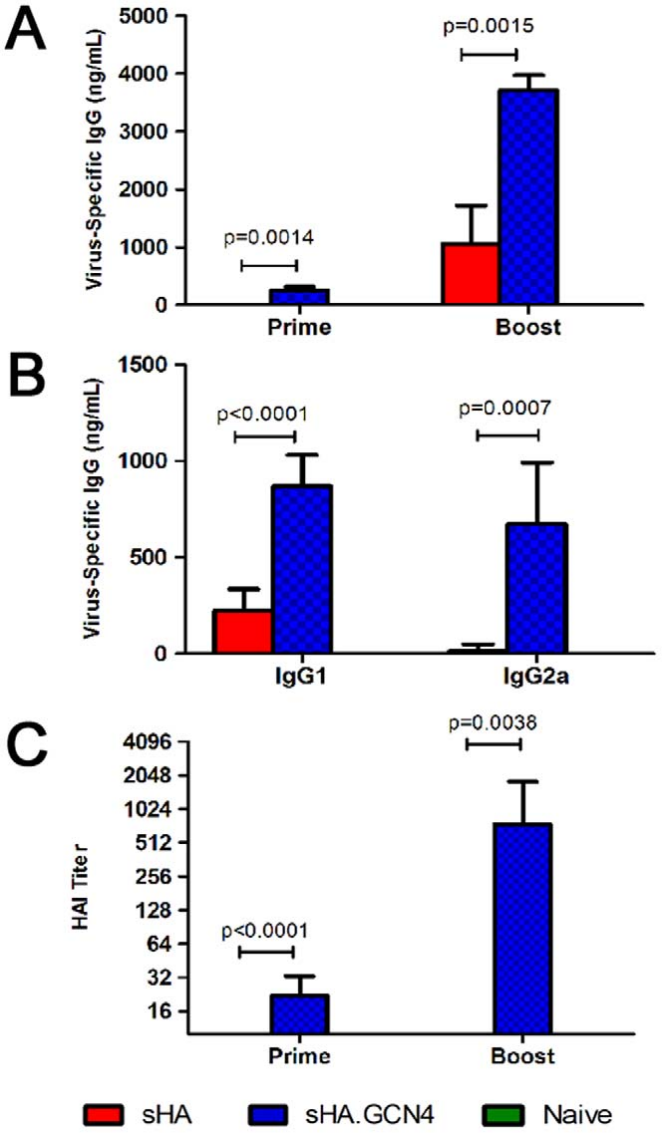
sHA.GCN4pII induces more robust humoral responses compared to sHA. **A.** Mice were primed and boosted 3 weeks later with 3 µg of sHA (red), sHA.GCN4pII (blue) or PBS (naïve – green). Blood was collected day 21 after priming (prime) and 21 days after boosting (boost). ELISA plates were coated with 4 µg/mL of A/Aichi/2/68 virus and antigen specific serum IgG ELISA was measured for prime and boost. HRP-conjugated goat anti-mouse IgG was used for detection. **B.** IgG subtype ELISA. Sera collected 21 days after boosting was tested antigen-specific IgG subclass by ELISA as described in Materials and Methods. ELISA plates were coated with 4 µg/mL of A/Aichi/2/68 and antigen specific IgG subclasses were detected using HRP-conjugated goat anti-mouse IgG1 or IgG2a. **C.** Hemagglutination inhibition test of prime and boost sera was performed as described in Materials and Methods using live MDCK grown A/Aichi/2/68. (n = 6 per group)

To compare the immunogenicity of the trimeric sHA.GCN4pII and the unmodified sHA, female Balb/c mice (6–8 weeks) were vaccinated subcutaneously (s.c.) with 3 µg of sHA or sHA.GCN4pII at day 0 and boosted at day 21. Mice primed with the sHA.GCN4pII had serum IgG responses to homologous inactivated A/Aichi/2/68 virus while mice primed with sHA had no detectable antigen-specific IgG by ELISA (Figure 4A). Following boosting, the sHA.GCN4pII vaccinated group had approximately 15-fold higher serum IgG titers than the sHA vaccinated mice (Figure 4A).

The HAI test is used to measure antibodies which bind to the HA receptor binding domain, blocking binding to sialic acid receptors. In general, an HAI titer of ≥40 is correlated with vaccine-induced protection in humans[22]. Therefore, serum collected from mice primed and boosted with the recombinant proteins were tested for HAI titers. At 21 days after priming, the sera from sHA.GCN4pII vaccinated mice had a geometric mean HAI titer of 16 while the sera from the sHA vaccinated group had no detectable HAI activity. Following boosting, sera from mice vaccinated with sHA.GCN4pII had a geometric mean HAI titer of approximately 700 while the sHA group had no detectable HAI titer (Figure 4C). Thus, the modified trimeric H3N2 sHA vaccine has a significantly enhanced ability to induce functional anti-influenza antibodies compared to the unmodified H3N2 sHA antigen.

### Mice vaccinated with sHA.GCN4pII express similar levels of serum IgG1 and IgG2a

The differences in biological functions of the IgG subclasses are well known [23] and the helper T cell cytokines responsible for class switching have been studied extensively [24]. Therefore, we compared the antigen specific IgG1 and IgG2a levels in mice vaccinated with sHA or sHA.GCN4pII by ELISA. On day 21 after boosting, mice vaccinated with sHA.GCN4pII had higher serum levels of antigen specific IgG1 than mice vaccinated with sHA. In addition, mice vaccinated with sHA.GCN4pII had antigen specific serum IgG2a while the mice vaccinated with sHA had no detectable antigen specific IgG2a (Figure 4B). For the sHA.GCN4pII vaccinated mice, similar relative amounts of IgG1 and IgG2a were observed suggesting a balanced Th1/Th2 cytokine environment.

### sHA.GCN4pII induces a protective immune response to lethal challenge with mouse adapted A/Aichi/2/68 virus

**Figure 5:**
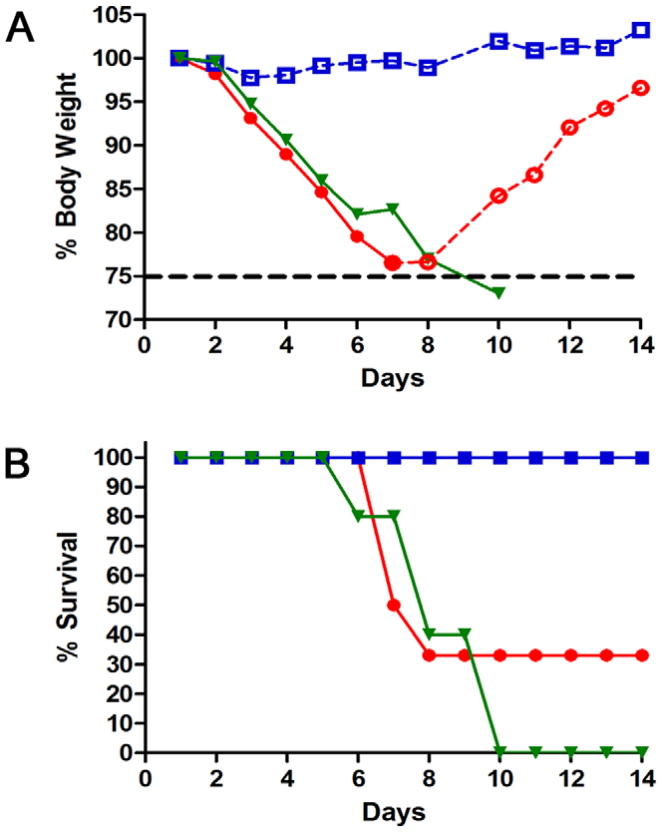
sHA.GCN4pII provides complete protection after two vaccinations unlike sHA. Mice primed and boosted were challenged with 5 xLD_50_ of mouse adapted H3N2 A/Aichi/2/68. **A, B.** Body weights and survival were followed for 14 days post challenge. sHA (circle), sHA.GCN4 (square), PBS (inverted triangle). Open symbols with dashed line indicate % mean initial body weight of surviving mice for each group. Closed symbols with solid line indicate % mean initial body weight of mice showing signs of morbidity. (n = 6 per group)

To compare the efficacy of the trimeric sHA and the monomeric sHA as vaccine antigens, mice vaccinated with 3 µg of sHA or sHA.GCN4pII were challenged with 5 x LD50 of homologous mouse adapted A/Aichi/2/68 virus by intranasal instillation. Body weights and survival were monitored for 14 days post challenge. Following challenge, the sHA group had an average body weight loss of approximately 20% at a rate comparable to the PBS treated (naïve) mice (Figure 5A). However, the sHA.GCN4pII vaccinated group maintained their initial body weight for the duration of the experiment. Notably, mice that received the sHA.GCN4pII vaccine had a survival rate of 100% (5/5) compared to mice vaccinated with sHA which had a survival rate of 33% (2/6) (Figure 5B)(p = 0.0269). This difference in weight loss and survival demonstrates the enhanced efficacy of the modified trimeric Aichi GCN4pII HA vaccine compared to the unmodified H3N2 HA vaccine.

### A single immunization with sHA.GCN4pII increases survival and decreases signs of morbidity

**Figure 6:**
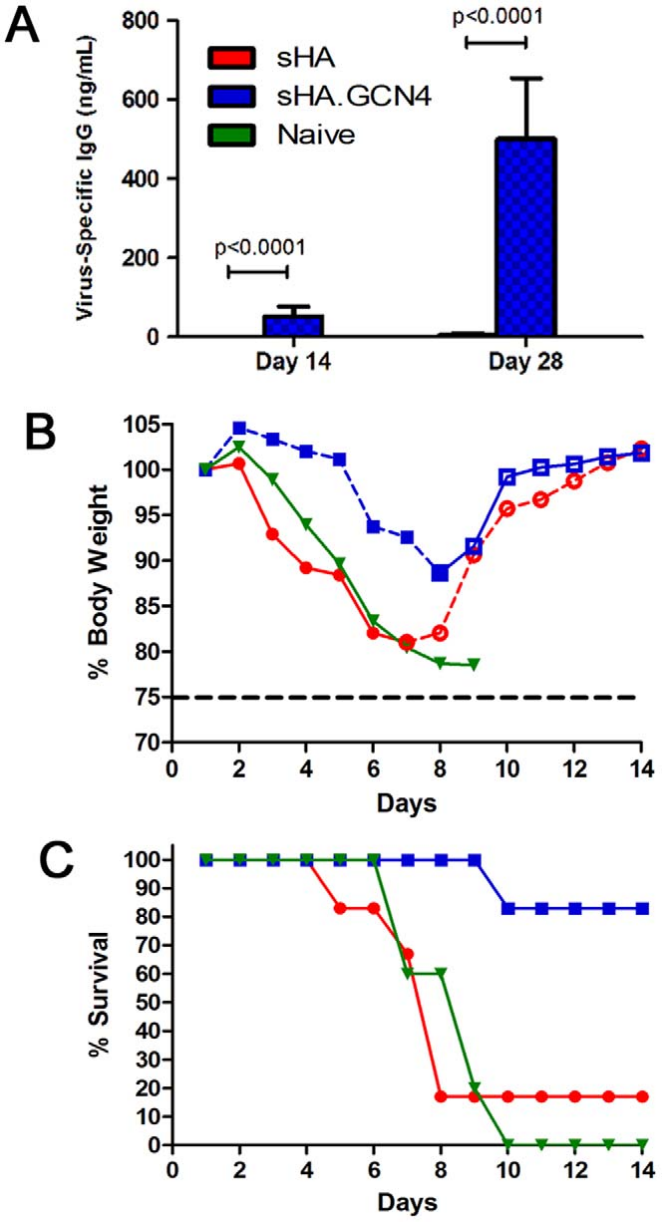
sHA.GCN4pII induces stronger protective immune response after a single vaccination compared to sHA. **A.** Serum IgG ELISA. Plates were coated with 4 µg/mL of A/Aichi/2/68 and antigen specific IgG was detected in sera collected on day 14 and day 28 post-vaccination using HRP-conjugated goat anti-mouse IgG. Mice vaccinated with recombinant Aichi HA proteins were challenged on day 31 after vaccination with 5 x LD50 of mouse adapted A/Aichi/2/68. **B, C.** Body weights and survival were followed for 14 days post challenge. sHA (circle), sHA.GCN4 (square), PBS (inverted triangle). Open symbols with dashed lines indicate % mean initial body weight of surviving mice for each group. Closed symbols with solid lines indicate % mean initial body weight of mice showing signs of morbidity. (n = 6 per group)

To determine the efficacy of a single vaccination, female Balb/c mice (6–8 weeks) were vaccinated s.c. with 3 µg of sHA or sHA.GCN4pII. Serum was collected on days 14 and day 28 post-vaccination and serum IgG and HAI titers were measured as above. On day 14, sHA.GCN4pII induced a virus-specific serum IgG response of approximately 52 ng/mL while sHA induced no detectable virus specific IgG. On day 28 following vaccination, sHA.GCN4pII induced a 144-fold higher virus-specific serum IgG response compared to animals vaccinated with sHA (Figure 6A). Two of six animals from the sHA.GCN4pII group were positive for HAI titer while sHA vaccinated mice had no detectable HAI titer (data not shown).

Mice were challenged with 5 x LD_50_ of mouse adapted A/Aichi/2/68 by intranasal instillation. sHA.GCN4pII vaccinated mice had limited body weight loss compared to sHA vaccinated mice or control mice (Figure 6B). Despite modest IgG and HAI titers, 5/6 of the sHA.GCN4pII vaccinated mice survived the challenge compared to sHA vaccinated mice in which only 1/6 survived (Figure 6C). These results demonstrate that stabilization of the trimeric structure of the H3N2 soluble HA enhances protective immune responses following a single vaccination in the absence of adjuvants (p = 0.0195).

## Discussion

Influenza virus remains an important respiratory pathogen with the potential to cause worldwide pandemics, such as the “Spanish” influenza virus in 1918 and the novel swine-origin H1N1 pandemic in 2009 [25]. Furthermore, the recent delays in the production of the monovalent H1N1 vaccine illustrate the need for a rapidly produced and safe alternative to egg-based influenza vaccines. Here we investigated the use of recombinant baculovirus expression to produce soluble forms of the HA protein, which is the primary target of neutralizing antibody. This system has several advantages including the high levels of protein expression [6] and an established safety record in humans[2], [5], [7]. Current influenza vaccine production requires the generation of a high-yield egg growth reassortant to obtain a sufficient yield for vaccine production. Using molecular cloning, the HA gene from circulating viruses can be cloned directly from clinical isolates and a recombinant protein vaccine can be rapidly produced to prevent new seasonal outbreaks or pandemics as a result of antigenic drift or shift [2], [8].

In this study, we generated a rBV-derived soluble hemagglutinin (sHA) modified at its C-terminus with the GCN4pII trimerization heptad repeat to stabilize the trimeric structure of the secreted protein. The heptad repeat is derived from the yeast transcription factor GCN4, which plays a role in the biosynthesis of amino acids during starvation. The wildtype heptad repeat forms stable dimers; however by mutating the ‘a’ and ‘d’ positions in the heptad repeat, trimeric or tetrameric coiled coils can form [16], [17]. This strategy has been used recently to study the structure and function of the HIV-1 envelope glycoprotein and parainfluenza virus 5 F protein [26], [27].

The sHA selected for this study was derived from the H3N2 virus A/Aichi/2/68 due to the extensive data on its structure and function previously published [12], [28], [29], [30]. Our data indicated that the GCN4pII modification stabilized the trimeric sHA in solution as determined by BS3 crosslinking and analysis by Western blot. Mice primed and boosted with a low dose of recombinant sHA.GCN4pII without an adjuvant had an improved humoral response to the H3N2 HA compared to mice receiving the unmodified sHA protein. Our results are also consistent with results using the H3N2 virus A/Victoria/3/75 soluble HA in which the monomeric HA induced HA specific antibodies; however there was no detectable binding to the trimeric viral HA[31]. In addition, mice vaccinated with the A/Victoria/3/75 sHA were not protected against challenge with homologous mouse adapted virus.

Other studies using recombinant influenza sHA have used HA from H1 [4], [5], [8], H3 [3], [5], [8], [32], [33], H5 [10], [21], [34], and H7 [21] subtypes in addition to influenza B hemagglutinin [5], [8] as a vaccine antigen. The properties of these sHA have been analyzed using hemagglutination assay, trypsin susceptibility, and/or electron microscopy [4], [7], [21]. Overall, the ability of these recombinant proteins to form stable trimers varies among subtypes and viruses. In the current study, we have employed a water-soluble crosslinker, BS3, which links monomers within an oligomeric structure to characterize the trimeric structure of the recombinant H3 sHA [19], [20]. Crosslinking provides the advantage of being able to determine the approximate ratio of oligomers in solution without the need to use complex size-exclusion chromatography techniques. Modification of the H5 HA with the foldon trimerization domain from the T4 fibritin protein also was reported to generate soluble trimeric HA in the recombinant baculovirus system which was more immunogenic than the unmodified protein when mice were vaccinated with recombinant protein with adjuvant [10]. In addition, the GCN4 trimerization repeat has been used to study the immunogenicity of H5 HA in poultry[35]. Our data clearly demonstrate that the enhanced immunogenicity of the trimeric H3 HA is due to the presentation of native trimeric epitopes in the GCN4pII stabilized protein, while the unmodified protein presents epitopes associated with the altered low-pH conformation of the HA protein.

Our data indicate that sHA.GCN4pII generates higher levels of both IgG2a and IgG1 than the soluble Aichi HA. The ratio of IgG1:IgG2a that we observe following boosting suggests that the cytokine environment is a balanced Th1/Th2 phenotype in the sHA.GCN4pII group while the mice immunized with sHA exhibit a Th2 phenotype. In the influenza mouse model, virus specific class 1 helper T cells (Th1) have been suggested to play a more important role in viral lung clearance compared to class 2 helper T cells (Th2) which are associated with increased airway inflammation and enhanced morbidity[36]. Furthermore, the higher serum levels of IgG2a, which is regulated by Th1-associated cytokines[37], in sHA.GCN4pII vaccinated mice might suggest that the finding of more efficient clearance of virus could be related to the effector functions of IgG2a such as complement activation and Fc receptor binding[23], [38], [39]. Therefore, both the quantity and quality of the humoral immune response in addition to the helper T cell phenotype should be considered in evaluating influenza vaccine efficacy.

It is noteworthy that the protection observed did not require the use of any adjuvants in the vaccination, although we cannot exclude any undocumented adjuvant properties of the GCN4pII repeat. Using the modified recombinant HA, we were able to induce complete protection in mice following a lethal challenge with mouse adapted A/Aichi/2/68 after two vaccinations. Furthermore, we were able to provide protection to 5/6 of mice following a single vaccination with 3 µg of sHA.GCN4pII in the absence of adjuvants. Thus, the trimeric antigen has a high potential to induce protective immune responses with just a single vaccination, which may be further enhanced by including approved adjuvants.

These results demonstrate that using the expression construct employed in this study, the instability of HA is eliminated by stabilizing the trimeric structure through the GCN4pII trimerization heptad repeat. Furthermore, the stabilized trimeric HA has been demonstrated to exhibit enhanced immunogenicity over unmodified HA with just one vaccination in the absence of adjuvants. These differences could be explained by the presence of the low-pH conformation in the unmodified sHA detected by monoclonal antibody binding. Previous studies on the structure of the X31 HA (A/Aichi2/68) monomer indicate that the trimeric protein is dissociated into monomeric subunits[40]. Antibodies that recognize this low pH structure might interact with epitopes at the monomer-monomer interface, or the “silent face”, of the hemagglutinin molecule. By presenting the “silent face” in the unmodified sHA, the antibody repertoire maybe skewed to epitopes not accessible in live virus at neutral pH. Therefore, by conserving the native trimeric structure of the HA, the sHA.GCN4pII represents a better influenza subunit vaccine candidate.

## Materials and Methods

### Ethics Statement

Mice were sterile housed and treated according to Emory University School of Medicine (Atlanta, GA) guidelines and all animal studies were approved by the Emory University Institutional Animal Care and Use Committee.

### Generation of a soluble influenza hemagglutinin

The HA gene from A/Aichi/2/68 was obtained from David Steinhauer. The soluble HA (sHA) gene was generated by introducing a stop codon upstream of the transmembrane domain by PCR (K528). The sHA gene was cloned (Quickligase, New England Biolabs – Ipswich, MA) into the pET22b(+) vector (Novagen/Merck KGaA, Darmstadt, Germany) using BamHI and SalI to introduce a 6X His tag at the C-terminus and then subcloned into the recombinant baculovirus vector pFastBac1 (Invitrogen - Carlsbad, CA - Carlsbad, CA) using BamHI and XbaI. The DNA segment encoding the trimeric GCN4pII heptad repeat (RMKQIEDKIEEILSKIYHIENEIARIKKLVGER) derived from the wildtype dimeric GCN4 repeat found in *Saccharomyces cerevisiae* was constructed using two rounds of PCR then cloned into pET22b(+)[16], [17]. The GCN4pII sequence was then subcloned into pFastBac1containing the sHA gene using EagI and SalI as described above (sHA.GCN4pII). All plasmids were sequenced to confirm deduced amino acid sequences (Agencourt/Beckman Coulter Genomics – Danver, MA).

### sHA and sHA.GCN4pII recombinant baculovirus production

pFastBac1 vectors containing the sHA and sHA.GCN4pII genes were transformed into DH10Bac cells and recombinant baculoviruses were generated according to the manufacturer's protocol (Invitrogen - Carlsbad, CA). Sf9 cells were transfected with bacmids as previously described [41].

### Recombinant sHA protein expression and His-tag affinity purification

Plaque assays were performed according to the Bac-to-Bac Kit protocol (Invitrogen - Carlsbad, CA). Sf9 cells were infected at a MOI of 1 and incubated for 48 hours to express recombinant protein. Supernatants were collected, clarified by centrifugation then affinity purification was performed by incubating recombinant proteins overnight at 4°C with nickel-agarose beads (Qiagen – Valencia, CA) and washing in empty columns and to remove rBV and cellular proteins. Non-specific nickel-bead binding was eliminated by washing with 10 mM imidazole (Sigma – St. Louis, MO) and recombinant protein eluted with a 250 mM imidazole solution. Protein elution was monitored using a UV detector at 280 nm. Imidazole was removed by dialysis against PBS (Gibco - Carlsbad, CA).

Purified proteins were separated on a 10% SDS-PAGE under reducing conditions (1% mercaptoethanol) then blotted and analyzed by Western blot using anti-A/Aichi/2/68 serum, anti-6xHis monoclonal antibody (Qiagen – Valencia, CA) and Coomassie blue stain (Biorad – Hercules, CA). Western blot was developed using the substrate ECL-Plus (BioRad – Hercules, CA).

### Bis[sulfosuccinimidyl] suberate (BS3) crosslinking

The oligomeric status of purified recombinant proteins was determined using the water soluble BS3 crosslinker (Pierce - Rockford, IL). Crosslinking was performed as described by De Fillette et al [42], with the following modifications. Briefly, 1 µg recombinant protein was incubated at room temperature in the presence of BS3 (final concentration – 3 mM) for 30 minutes. Crosslinking was stopped by the addition of 1M Tris-HCl pH 8.0 to a final concentration of 50 mM. After crosslinking, proteins were separated on a 5–15% SDS-PAGE under reducing conditions (1% mercaptoethanol) then blotted and analyzed by Western blot using anti-6xHis antibody and developed using ECL-Plus.

### Sandwich ELISA

Sandwich ELISAs were performed as described by Vanlandschoot et al [31], [33], with modifications. Briefly, 96-well Immunoplates (Nunc) were coated with guinea pig anti-A/Aichi/2/68 polyclonal sera (0.02 µg/mL)(BEI Resources). After three washes (0.05% Tween-20 in PBS), wells were blocked with 3% BSA in wash buffer for 2 hours at 37°C, followed by three washes. Recombinant proteins were added to each well (20 ug/mL in PBS) and incubated for 1.5 hours at 37°C, followed by three washes. To determine antibody binding, 2-fold serial dilutions (PBS) of anti-influenza A/Aichi/2/68 sera (mouse), LC89, or HC67 (a kind gift of David Steinhauer) were added and incubated for 1.5 hours at 37°C, followed by three washes. Wells were incubated with goat anti-mouse IgG conjugated to horseradish peroxidase (1∶500 in PBS) for 1.5 hours at 37°C, followed by three washes. OPD substrate was added to each well and color allowed to develop. Color development was stopped using 1 M phosphoric acid. Absorbances were read at 450 nm on a Biorad Model 680 microplate reader.

### Vaccination and Serological Immune Responses

Female Balb/c mice (6–9 weeks old)(Harlan Laboratories) were lightly anesthetized with a mixture of xylazine and ketamine and vaccinated subcutaneously with 3 µg of sHA, sHA.GCN4pII, or PBS (naïve) on day 0 and day 21. Mice were then retro-orbitally bled on d21 and d42 and serum collected for hemagglutination inhibition tests (HAI) and Ig ELISA.

HAI tests were performed on vaccinated animal sera based on the WHO protocol [43]. Briefly, sera was treated with receptor destroying enzyme (Denka Seiken Co. Ltd, Tokyo, Japan) for 16 hours at 37°C then heat inactivated for 30 minutes at 56°C. Treated sera was diluted to a final concentration of 1∶10 in PBS and incubated with pack chicken RBC for 1 hour at 4°C to remove cryoglobulins. Treated sera were serially diluted and incubated with 4 HA units of A/Aichi/2/68 virus for 30 minutes at room temperature. An equal volume of 0.5% chicken RBC was added to each well and incubated for 30 minutes at room temperature. The HAI titer was read as the reciprocal of the highest dilution of serum that inhibited hemagglutination. Values were expressed as the geometric mean with a 95% confidence interval.

### Virus and challenge

To determine vaccine efficacy, vaccinated mice were lightly anesthetized with isofluorane and challenged by intranasal inoculation of 50 µL with 5 x LD50 of live mouse adapted A/Aichi/2/68. Body weight loss and survival rates were monitored daily for 14 days post challenge. Weight loss ≥25% was used as end-point and mice were euthanized according to IACUC guidelines.

### Statistical analysis

Statistical analysis was done using the one-way ANOVA analysis of grouped data with GraphPad Prism5 software. Survival curves were analyzed using the log-rank test. All data followed normal Gaussian distributions unless otherwise noted. A P value less than 0.05 was defined as a significant difference.
